# Neurodegenerative Proteinopathies Induced by Environmental Pollutants: Heat Shock Proteins and Proteasome as Promising Therapeutic Tools

**DOI:** 10.3390/pharmaceutics15082048

**Published:** 2023-07-30

**Authors:** Paula Moyano, Emma Sola, María Victoria Naval, Lucia Guerra-Menéndez, Maria De la Cabeza Fernández, Javier del Pino

**Affiliations:** 1 Department of Pharmacology and Toxicology, Veterinary School, Complutense University of Madrid, 28040 Madrid, Spain; jdelpino@pdi.ucm.es; 2 Department of Pharmacology, Pharmacognosy and Bothanic, Pharmacy School, Complutense University of Madrid, 28041 Madrid, Spain; 3 Department of Physiology, Medicine School, San Pablo CEU University, 28003 Madrid, Spain; 4 Department of Chemistry and Pharmaceutical Sciences, Pharmacy School, Complutense University of Madrid, 28041 Madrid, Spain

**Keywords:** environmental pollutants, heat shock proteins, proteasome, proteinopathies, neurodegenerative diseases

## Abstract

Environmental pollutants’ (EPs) amount and diversity have increased in recent years due to anthropogenic activity. Several neurodegenerative diseases (NDs) are theorized to be related to EPs, as their incidence has increased in a similar way to human EPs exposure and they reproduce the main ND hallmarks. EPs induce several neurotoxic effects, including accumulation and gradual deposition of misfolded toxic proteins, producing neuronal malfunction and cell death. Cells possess different mechanisms to eliminate these toxic proteins, including heat shock proteins (HSPs) and the proteasome system. The accumulation and deleterious effects of toxic proteins are induced through HSPs and disruption of proteasome proteins’ homeostatic function by exposure to EPs. A therapeutic approach has been proposed to reduce accumulation of toxic proteins through treatment with recombinant HSPs/proteasome or the use of compounds that increase their expression or activity. Our aim is to review the current literature on NDs related to EP exposure and their relationship with the disruption of the proteasome system and HSPs, as well as to discuss the toxic effects of dysfunction of HSPs and proteasome and the contradictory effects described in the literature. Lastly, we cover the therapeutic use of developed drugs and recombinant proteasome/HSPs to eliminate toxic proteins and prevent/treat EP-induced neurodegeneration.

## 1. Environmental Pollutants and Neurodegenerative Diseases

One of the most pressing health-related issues that societies face at present is the increase of non-transmittable diseases such as neurodegenerative diseases (NDs), as their incidence has increased exponentially in the last decades. In parallel, contaminants released into the environment have increased in both quantity and compound classes, leading to pollution and deleterious effects on living beings. Environmental pollutants (EPs) have been pointed out as one of the possible etiological factors of NDs, even though the complete causes that mediate their induction remain unclear. This is supported by the parallel increase in contaminants (toxic metals, pesticides, industrial/commercial pollutants, antimicrobials, and air pollutants, among others) released into the environment and the incidence of NDs, as well as by the fact that these compounds have been found to induce the main hallmarks and symptoms of several NDs in both in vitro and animal studies [1,2,3,4,5]. Moreover, experimental data and epidemiological studies have suggested that there is an important risk of developing NDs due to EP exposure [6].

In this regard, several environmental contaminants, such as metals including cadmium (Cd), manganese (Mn), aluminum (Al), and lead (Pb), among others, produce neurotoxic effects and have been linked to the generation of various NDs [1,2,3,4,5,6,7,8,9,10,11]. Pesticides such as organophosphate and pyrethroid insecticides, including deltamethrin (DM), and herbicides such as paraquat (PQ) and glyphosate (GP), among other biocides, have been shown to induce neurodegeneration and cognitive impairment [12,13,14,15,16,17]. In murine neonates exposed to brominated flame retardants (PBDEs) such as congener 47 (PBDE-47) the induction of behavioral changes has been observed, whereas studies in adult mice have shown learning and memory impairment [18]. Plasticizers such as bisphenol A (BPA) and phthalates cross the fetoplacental barrier and have been found to cause growth retardation and neurological damage as well as the induction of cognitive alterations in adulthood [19]. Broad-spectrum antimicrobials such as triclosan (TCS), which are active ingredients in many consumer products, including soaps and toothpaste, have been reported to cause neurodevelopmental and behavioral changes along with impaired learning and memory processes [20,21].

**Figure 1:**
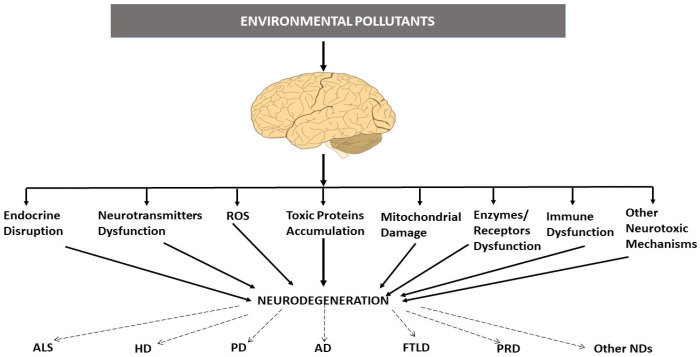
**EP-induced neurotoxic mechanisms associated with NDs.** Mechanisms through which EP exposure may be able to induce neurodegeneration that may lead to the development of NDs. Solid line arrows indicate mechanisms and neurotoxic effects shown in the literature, while dashed line arrows indicate suggested associations.

The brain regions in which EPs accumulate are the ones in which neurodegenerative events leading to NDs occur; this, together with the fact that EPs share several mechanisms hypothesized to induce them, supports the involvement of EPs in ND induction (Figure 1) [1,3,4,12,15,16,17,22]. The presence of EPs in the brain has been observed to induce toxic protein accumulation and aggregation, mitochondrial damage, cell signaling impairment, inflammation, reactive oxygen species (ROS) generation, dysregulation of different enzymes and receptors, and neurotransmission alteration, among others, leading to neuronal malfunction and loss, which in turn results in movement and cognitive disruption, among other dysfunctions [1,3,4,12,15,16,17,22].

New drug research for ND treatment is an arduous task which has led to few favorable discoveries. A common characteristic of most NDs is the induction of misfolded toxic protein that accumulate, leading to neuronal dysfunction and loss [23,24]. As stated, several EPs with diverse chemical compositions and structures have been suggested to induce the same NDs. Misfolded toxic proteins have been described being produced, accumulated, and deposited following EP exposure, triggering neurodegeneration [3,22,25,26]. Thus, this may explain how several EPs mediate the induction of specific NDs.

The refolding and degradation of toxic proteins are mediated by the proteasome system and the heat shock protein (HSP) family, among other mechanisms [27,28,29,30,31]. The family of HSP proteins have diverse functions during development, and can be found in all prokaryote and eukaryote living organisms [32]. In adults, HSPs are involved in protein homeostasis, cell signaling, the cell cycle, and apoptosis, among other tasks [33]. HSPs constitute 5–10% of expressed proteins, and become overexpressed under stress situations as a defense mechanism [34]. There are different HSP categories depending on molecular weight, including the HSP110, HSP100, HSP90, HSP70, HSP60, HSP40, and small HSP families [35]. Their transcription is controlled by the action of heat shock transcription factor 1 (HSF1), which induces their upregulation when it attaches to the heat shock elements (HSE) in the promoter of HSP genes [36,37,38]. They can be found both intra- and extracellularly [39,40]. HSPs bind to newly synthesized proteins, folding them correctly and scanning proteins to locate aberrant or misfolded ones which are wrongly folded or aggregated in order to repair or disaggregate them. When they cannot be repaired, HSPs induce the proteasome or autophagy pathways, which conform to mechanisms of degradation [26,41].

The proteasome 26S (P26S) complex, consisting of proteasome 20S (P20S) and one or two proteasome 19S (P19S) particles, mediates the removal of ubiquitinated proteins through the ubiquitin–proteasome system (UPS) [30,31]. The proteasome 19S (which recognizes target proteins and deubiquitinates, then unfolds and delivers them to the 20S) and proteasome 20S are the regulatory particle and the catalytic core, respectively, of the proteasome 26S [30,31]. The proteasome 20S alone, independently of the ubiquitination pathway, mediates the degradation of misfolded and damaged proteins, presenting different variants depending on the tissue, cells, and probably function [30,31]. P19S, which contains many ATPase active sites, attaches to either one or both sides of the 20S core particle [42]. The ATPases sites using ATP energy unravel the folded protein into lineal structures and insert them inside the P20S for breakdown [43,44,45]. The P20S is made up of four heptameric rings (α_7_β_7_β_7_α_7_), presenting catalytic activity in the β1, β2, and β5 subunits (caspase, trypsin, and chymotrypsin activities, respectively) [30]. The proteasome is necessary to maintain cell homeostasis [30,31]. In this regard, it is involved, in the regulation of neuronal transmission, the cell cycle, oxidative damage, immunomodulation, degradation of misfolded proteins, and translational modifications, among other actions [30,31]. These proteins are the most important system responsible for the elimination of body proteins, especially the misfolded and/or aggregated proteins, preventing the induction of cell damage [30,31].

EP exposure has been shown to alter HSP and/or proteasome protein expression and to disrupt their homeostatic function, resulting in accumulation of toxic proteins as well as cellular damage and loss [25,46,47,48,49,50,51,52,53]. Thus, therapeutic approaches to treat the neurodegeneration induced by the toxic proteins could include treatment with compounds that mediate the induction of HSP or proteasome protein expression to increase their activity and administration of recombinant HSPs or proteasome proteins.

## 2. Neurodegenerative Proteinopathies and Environmental Pollutants

Ribosomal protein formation, correct folding, delivery, post-translational modifications, and removal, which together make up proteostasis, may be altered by several factors. These alterations lead to changes in peptide conformation and tridimensional shape, triggering their aggregation and precipitation in the form of toxic proteins [25,26]. The accumulation of aberrant proteins leads to impairments in cell functions and ultimately to their loss [25]. Toxic proteins have been described as accumulating in several NDs, including Alzheimer’s Disease (AD), Amyloid Lateral Sclerosis (ALS), Huntington’s Disease (HD), Frontotemporal Dementia (FTLD), Parkinson’s Disease (PD), and Prion disease (PRD), among others [3,22,23,24,25,26,41]. Although, there are no specific toxic proteins that are exclusive of any particular ND, several may be present in different NDs [25,41]; for example, amyloid-beta (Aβ) and hyperphosphorylated-Tau (p-Tau) have been described as accumulating in AD, alpha-synuclein (α-syn) in PD, transactive response DNA binding protein 43 (TDP-43) in FTLD and ALS, huntingtin protein (HTT) in HD, and prion proteins (PrP) in PRD [25,41].

EPs are associated with the formation and accumulation of these toxic proteins. Alpha-synuclein accumulation has been described as being induced by several compounds after repeated exposure, such as PQ in mice striatum [54], rotenone in rat ventromedial midbrain [55], arsenic in mice striatum and cortex [56], atrazine in CD1 mice brain [57], and ochratoxin A in mice brain [58], among others, or after single treatment, such as diethyldithiocarbamate in rat hippocampal astrocytes [59] and Mn in SH-SY5Y cells [60], among others. Moreover, single and repeated exposure to cadmium, manganese, and chlorpyrifos in SN56 cells from basal forebrain (BF) and paraquat in primary hippocampal neurons have been associated with the accumulation of Aβ and p-Tau proteins, among others [7,49,51,52]. In the same way, dioxins (in BE-M17 cells, motor neuron cells, and in C57Bl/6 J mice brain), lead (in PC12 cells, primary hippocampal neurons, and in BALB/c mice), mercury (in PC12 cells, primary hippocampal neurons, and in BALB/c mice), and paraquat (in SH-SY5Y cells) have been reported to induce the accumulation of TDP-43 [61,62,63]. HTT has been described as aggregating following 3-nitro-propionic acid, paraquat, dieldrin, and rotenone exposure, among others [64], while cadmium was described as inducing the production of prion proteins in PC12 cells and α-terthienyls as triggering their aggregation [65,66].

Refolding into the correct shape, or the induction of protein degradation when the first is not possible, are among the mechanisms that cells have to avoid the harmful effects of toxic proteins. The unfolded protein response (UPR) in the endoplasmic reticulum (ER) and the heat shock response (HSR) in the cytosol are systems induced to avoid the accumulation of misfolded toxic proteins in these two cell compartments [67,68]. UPR activation transitorily reduces protein synthesis and induces the upregulation of different proteins that mediate the refolding of these toxic proteins in the ER through the action of PKR-like endoplasmic reticulum kinase (PERK) and inositol-requiring 1α (IRE1 α) and the activation of transcription factor 6 (ATF6), which are bound to the chaperone HSPA5, known as glucose regulating protein 78 (GRP78) or binding immunoglobulin protein (BiP), and released when toxic proteins bind to BiP [67,68,69,70]. First, chaperones try to refold the accumulated misfolded proteins; in the cases where they are heavily damaged, they are sent for degradation mediated by the endoplasmic reticulum-associated degradation (ERAD) pathway through the proteasome or autophagy systems [67,68,69,70].

**Figure 2:**
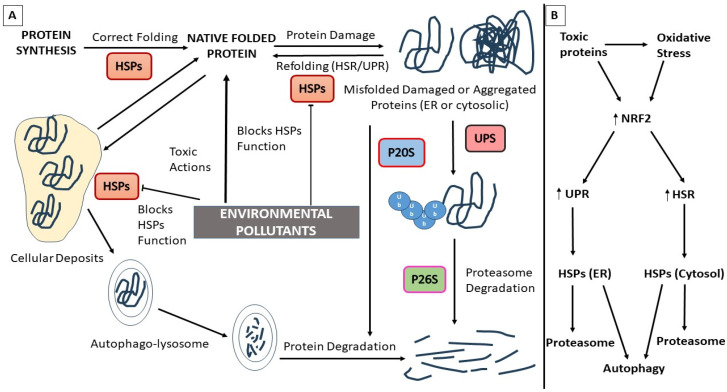
**Toxic protein degradation mechanisms.** EP-induced stress leads to the formation and aggregation of aberrant misfolded toxic proteins (**A**). Toxic protein accumulation upregulates NRF2, which can induce the UPR or HSR systems to activate for their degradation (**B**). Cells induce HSP overexpression to refold these proteins when the damage is not extensive. When toxic proteins cannot be repaired, cells induce their degradation through the proteasome, autophago-lysosome, or their accumulation within transient or stable cellular deposits before degradation. Solid line arrows indicate mechanisms shown in the literature.

HSR system action is regulated by HSF1, which is united to HSP90 in the cytosol; when the misfolded proteins accumulate, they unite with HSP90 in releasing HSF1, leading to the upregulation of cytosolic HSPs in order to refold these toxic proteins accumulated in the cytosol. When these proteins are largely damaged, UPS is induced by the chaperones in order to produce protein breakdown [27,28,29,67,68]. Chaperone-mediated autophagy (CMA) is a different mechanism that allows protein degradation in which the action of the lysosomal-associated membrane transporter (LAMP2a) is mediated by HSPs, introducing misfolded proteins inside the lysosome [71,72,73]. If the degradation pathways do not function, toxic proteins can await their degradation within transient or stable cellular deposits (Figure 2A) [41]. Accumulation of toxic proteins is induced when these clearance mechanisms are disrupted, as observed following exposure to EPs or in NDs [26].

Nuclear factor erythroid-derived 2-like 2 (NRF2) is a transcription factor that regulates cellular response against oxidative stress. Under no-stress situations, kelch-like ECH-associated protein 1 (KEAP1) is bound to NRF2, leading to ubiquitination and elimination by proteasome action; however, when oxidative stress is induced, KEPA1 releases NRF2, which translocates to the nucleus. Here, it induces the expression of several target genes that mediate the antioxidant response, including HSF1, HSPs, autophagy, proteasome subunits, and UPR pathway target genes, among others (Figure 2B) [74]. Thus, NRF2 is an important regulator of UPR and HSR through the proteasome and autophagy systems.

## 3. Heat Shock Proteins (HSPs) and EPs

Environmental pollutants (metals, herbicides, pesticides, fungicides, and industrial chemicals, among others) have been related to proteinopathy generation and detrimental effects on the expression and function of HSPs [3,6,7,14,17,22,25,49,50,52,53,61]. However, few studies have established a correlation between both events and proven that the HSP dysfunction induced by EPs is the cause of toxic protein accumulation. The aim of this section is to discuss this correlation, its causality, and the mechanisms that mediate it.

### 3.1. Environmental Pollutants and HSP Dysfunction

Toxic proteins have been described as inducing ROS generation and initially increasing NRF2 levels; however, in later stages they produce downregulation of the NRF2 pathway [74,75]. Thus, the formation of aberrant toxic proteins could trigger the mechanisms that mediate their accumulation and aggregation. EP exposure has been reported to alter the NRF2 pathway [76,77,78,79,80,81,82,83,84,85,86,87,88,89,90,91,92,93]. Therefore, HSP dysfunction induced by EPs could be mediated through the formation of toxic proteins that disrupt the NRF2 pathway, and finally the action of HSPs.

Different research works have explored the effects of EPs on proteinopathies and HSP disruption. In this regard, exposure to the essential metal manganese, which is used in several industrial applications, leads to the aggregation of α-synuclein protein [94], increases the levels of p-Tau and Aβ [49,95,96], and promotes the conglomeration and stabilization of PrP [97]. One-day exposure to Mn (from 25 µM) induces the overexpression of HSP90, HSP70, and NRF2 proteins in BF SN56 cholinergic cells; although concentrations of over 50 µM after one-day exposure or after 14 days exposure from concentrations of 25 µM induces the downregulation of these proteins [49]. Acute exposure to low concentrations of Mn in spleen lymphocyte cells from chicken has been found to overexpress HSP70 and HSP90, whereas high concentrations downregulated their expression [98]. Moreover, the downregulation of HSF1 in the breast muscles of laying broiler breeders has been shown to be responsible for a decrease in the expression of HSP70 and HSP90 following Mn exposure [99]. The observed disruption of HSF1 could assist in explaining the effects described on HSPs regulated by this factor. NRF2 regulates HSPs [100,101], and the alteration of its expression in SN56 cells has been described as mediating the disruption in the expression of HSP70 and HSP90 [49]; thus, NRF2 may be able to mediate the described effects of Mn on HSPs.

Cadmium, an industrial heavy metal, induces α-synuclein protein aggregation [102] and increases p-Tau and Aβ protein levels [53]. Cd has been reported to induce downregulation of HSF1, HSP90, HSP70, and HSP27 in neuronal cells [53,103] and in different cell types from several species [104,105,106]. The reported downregulation of these HSPs was mediated by HSF1 downregulation in SN56 cells from BF [53]. Additionally, Cd downregulates the NRF2 pathway in BF neurons [76], which regulates HSF1 and HSPs expression. Thus, Cd could mediate the alteration of HSPs through NRF2 pathway disruption. However, the expression of these HSPs has been shown to increase in cells other than neurons from several species following Cd exposure [102,104,106,107,108]. In addition, Cd has been reported to induce the expression of other chaperones, such as αB-crystallin, HSP60, HSP40, and heat shock cognate 70 (HSC70), among others [105,109].

Arsenic (As), an industrial heavy metal, increases the levels of p-Tau and Aβ peptides [2,3] as well as α-synuclein proteins [56]. It was observed to induce the inhibition of neurofilament (NF) translocation, possibly leading to an abnormal distribution of NF in ALS [110]. Exposure to As has been described as inducing the expression of HSP27, HSP32, HSP60, HSP70, HSP90, and HSP110, among others, in both in vitro and in vivo models [47]. It has been reported that As induces NRF2 expression in MIHA cells [77], which could explain the observed HSP overexpression. Alternatively, As is able to eliminate the refolding capacity of HSPs as well as their ability to disaggregate misfolded proteins, which occurs because As can block the binding between HSPs and their substrates [111].

Mercury (Hg), present in both organic and inorganic compounds, has been shown to increase Aβ and p-Tau levels both in vitro and in vivo [3]. Inorganic mercury has been described as increasing the expression of HSP24, HSP70, and HSP90 in chicken embryos after 2 h and 4 h exposure [112] and of HSP70 in HeLa cells after 4 h and 7 h exposure [113]. In addition, inorganic mercury has been reported to induce HSP72, GRP94, and HSP73 in rat kidney after treatment for 4 h to 24 h, with differences in the disruption of these HSPs depending on the kidney region studied and the time of exposure [114]. Moreover, HSP70 was shown to be induced in workers who were involved in caustic soda production and chronically exposed to inorganic mercury [115]. Otherwise, methyl mercury (MeHg) treatment of HEK293 cells promotes the translocation of HSF1 into the nucleus, inducing overexpression of several HSPs, including HSPA1A and HSPA6 [116]. Conversely, HSP90 protein was downregulated following MeHg 12 h treatment in primary rat astrocytes [117]. Inorganic mercury exposure was reported to result in upregulated (in rat liver) or downregulated (in rat cardiac tissue) NRF2 levels [78,79], which could explain the opposite effect observed in HSP expression.

Aluminum, a heavy metal widely used in industry, has been observed to increase the content of Aβ and p-Tau proteins and induce their aggregation by increasing their synthesis and decreasing their degradation [3,118,119]. In addition, Al exposure has been found to lead to α-synuclein aggregation and the overexpression of the mutated toxic protein SOD-1 [2,3]. Al alters HSP expression, possibly leading to deleterious effects of aberrant misfolded proteins. In this regard, treatment of rats with Al for 48 days induced the overexpression of HSP70 in neurons and glial cells [120] and of HSP27 in primary human neural cells following treatment for 7 days [121]. In addition, treatment with Al for 6 months induced GRP75, HSP25, and HSP72 overexpression in rat liver and kidney [122]. Conversely, a 6 week treatment course with Al induced a downregulation of HSF1, HSP27, HSP40, and HSP70 expression and p-Tau and Aβ protein accumulation in the frontal cortex and hippocampus of rats [50]. Al exposure has been described as decreasing NRF2 levels in PC12 cells and in rat liver and kidney [80,81], which together with the downregulation of HSF1 could explain the effect observed on HSPs expression.

Paraquat, a widely used herbicide, and Maneb, an extensively used fungicide, have been described as increasing p-Tau and α-syn levels, and PQ, has been shown to increase Aβ levels [52,54]. Maneb and PQ have both been reported to increase HSP90 levels, and Maneb increased HSC70 expression and decreased HSP70 expression after intraperitoneal treatment twice weekly for six weeks in mice, though no effect on HSP70 and HSC70 expression was produced by PQ for similar exposure conditions in mice [54]. Maneb was reported to induce NRF2 pathway SH-SY5Y cells [82]. PQ was reported to upregulate NRF2 and HSP70 from 1 μM to 10 μM, while reducing their expression from 20 μM after 24 h of treatment and from 1 μM after 14 days of treatment in primary hippocampal cells [52,83]. Thus, PQ and Maneb effects on NRF2 could mediate the observed effect on HSP expression.

Atrazine, a widely used herbicide, has been related to the increase of α-synuclein and TDP-43 levels [57]. Atrazine treatment for 28 days decreased the expression of HSP70 and HSP90 in rare minnow female kidney, while HSP90 expression was downregulated and HSP70 was upregulated in rare minnow male kidney [123]. Atrazine treatment for 40 days induced HSP70 and HSC70 expression in common carp [124]. Atrazine has been described as inducing a dual effect on NRF2 expression, upregulating it at low doses and then starting to attenuate this upregulation at higher doses in quail kidney [84], which could explain the observed effect on HSP expression.

Bisphenol A (BPA), a chemical compound with multiple industrial applications, has been reported to induce Aβ and p-Tau accumulation [125]. BPA 24 h treatment was described to induce hormetic decrease of HSP6 and HSP70 expression (increase at low concentrations and decrease at higher concentrations); a decrease of HSP4 expression, and an increase of HSP16.2 expression in C. elegans [126]. BPA has been shown to decrease (in mice liver) and increase (in HEK293 cells) NRF2 expression [85,86], which could indicate mediation of the observed effects.

Perfluorooctanoic acid (PFOA), a perfluorinated compound with wide industrial applications, and was described to induce Aβ and p-Tau accumulation [127,128]. PFOA was reported to reduce the expression of HSPA5, and HSP27 after 72 h of treatment in L-02 cells in a concentration-dependent manner [129]. The reduction in NRF2 expression in mice testicles reported after PFOA exposure [87], could mediate the described reduction in HSPs.

Ochratoxin A (OTA), a fungal toxin, has been reported to increase the levels of α-synuclein [58]. OTA single treatment has been described as downregulating HSP70 and HSP27 protein levels in HepG2 cells and HSP70, HSP75, and HSP78 in Vero monkey kidney [130]. These effects were observed from 16 h of treatment at concentrations close to IC50 for cell viability (80 μM, respectively). Additionally, OTA was reported to upregulate hypoxia-inducible factor 1-alpha (HIF1α) (0.125 µM) after 24 h exposure and HSP90 expression (from 0.125 μM to 0.5 μM) after 24 h exposure. After 48 h exposure, the level was only at 0.125 μM in HEK293 cells, while HIF1α protein expression was downregulated after 48 h treatment (0.125 μM and 0.25 μM) [131]. OTA has been reported to downregulate NRF2 in porcine kidney tubule cells [88], supporting its participation in the alteration of HSPs expression.

Exposure to several insecticides has been related to the induction of proteinopathies, increasing the accumulation of toxic proteins including Aβ, p-Tau, α-syn, and HTT, among others [132,133,134,135,136]. Rotenone, an insecticide with neurotoxic effects similar to those observed in PD, has been reported to decrease HSP27 (striatum and substantia nigra), HSP90 (cerebellum, cortex, substantia nigra, and striatum) and HSP70 (cortex and substantia nigra) expression, while it induced an increase of HSP60 expression in the striatum, cerebellum, cortex, and substantia nigra after administration for 11 days to rats [137]. Rotenone treatment for 24 h has been shown to induce a reduction of HSP70 and HSC70 protein expression in SH-SY5Y cells from 0.4 µM concentration [133] and an increase of HSP70 expression from 1 µM concentration in SH-SY5Y cells [138]. Rotenone has been shown to downregulate (in mice striatum) and upregulate (in rat striatum) NRF2 levels [89,90], supporting the possible involvement of this factor in the regulation of HSPs expression observed.

Carbamates (formetanate, methomyl, pyrimicarb) and pyrethroids (bifenthrin) has been described as inducing GRP78 while downregulating HSP72/73 in A549 pulmonary cells after 3 days of treatment. Bifenthrin increased the expression of GRP94 and decreased the expression of HSP27, and methomyl downregulated HSP90 expression [139]. A study of the effects of organochlorines (dienochlor, endosulfan) and the neonicotinoid imidacloprid on the chaperones HSP27, HSP72/73, HSP90, GRP78, and GRP94 in the SH-SY5Y and A549 cell lines showed that after 3 days of treatment, these insecticides reduced the studied chaperones’ expression at concentrations higher than IC50 [140]. HSP27 was downregulated at concentrations of imidacloprid or endosulfan lower than IC50 [140]. GRP78 was upregulated by endosulfan in A549, but not in SH-SY5Y cells [140]. Conversely, HSP72/73 was found to be downregulated in both cell lines [140]. The organophosphate methyl-parathion was reported to downregulate HSP70 after 96 h exposure in zebrafish (Danio rerio) [141]. The avermectin eprinomectin, an insecticide and parasiticide, was shown to decrease HSP70 in liver tissue of rainbow trout exposed to sublethal concentration (0.001 μg/L, 0.002 μg/L, 0.01 μg/L, 0.05 μg/L) for 24 h, 48 h, 72 h, and 96 h [142]. Ivermectine, an avermectine insecticide and parasiticide, has been shown to inhibit HSP27 after single exposure in several tumoral cell lines [143]. Several of these insecticides’ effect on NRF2 have been determined, showing that they alter NRF2 levels [91,92,93] and supporting its possible mediation of HSP alteration.

**Figure 3:**
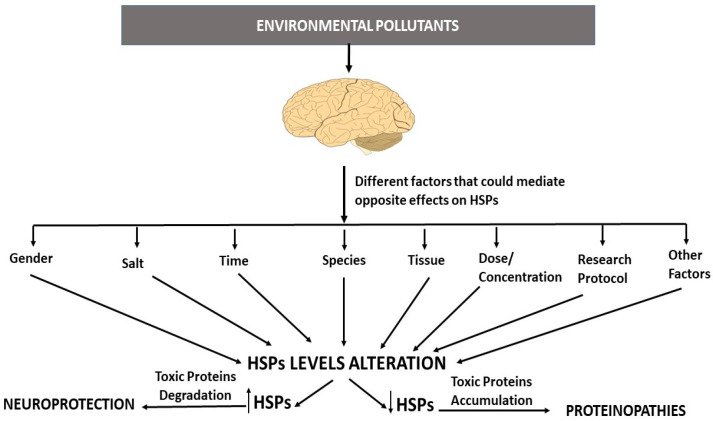
**EP factors that could mediate the differences in HSP expression.** The opposite effects of EPs on HSP expression shown in the literature and the possible factors that have been associated with the induction of these opposite effects. These factors may explain the neuroprotective or neurotoxic effects produced by HSP expression alteration induced after EP exposure. Solid line arrows indicate factor and neuroprotective/neurotoxic effects shown in the literature.

In addition to the NRF2 mediation of the differences reported in HSP expression following EP exposure, they may be explained by the diversity of several factors employed in the research, such as the use of different protocols, biological models, vehicles, or salts, as well as by gender differences (Figure 3) [144,145]. These reported differences may be related to the different times of exposure used in the studies, as indicated in the literature [106], and to the fact that initially HSPs are overexpressed to act as a defense mechanism against the stress produced immediately after EP exposure. At later stages, however, it is possible that additional toxic mechanisms of the EPs lead to HSP downregulation. It should be taken into consideration that these differences could be due to variations in the concentration/dose employed in the research, as different doses/concentrations may induce different stress levels. According to the literature, low stress levels produced after exposure to EPs at low doses/concentrations induce HSP expression, while high stress levels produced when concentrations/doses are increased can downregulate HSP expression, possibly due to ROS-induced denaturalization of HSF1, resulting in the inhibition of its function as a regulator of HSPs expression [106,146,147,148,149], although the induction of other toxic mechanisms that lead to HSP downregulation cannot be ruled out. Furthermore, HSP overexpression, initially produced due to EP-induced stress, could turn into decreased expression after the antioxidant mechanisms start to scavenge the generated ROS [150]. Finally, the observed differences could be related to opposite regulation of different HSPs, leading to some of them being induced and others being downregulated, as they can have diverse effects.

### 3.2. Heat Shock Protein Dysfunction, Neurodegenerative Proteinopathies, and EPs

The accumulation of toxic proteins leads to HSP induction with the aim of reducing their harmful effects. It has been reported that HSPs can help to avoid the conglomeration of Aβ proteins, one of the main hallmarks of AD, and induce the UPS, leading to the elimination of misfolded proteins [28,35,151]. Under pathological conditions, HSPs may fail in their role of protecting against the deleterious effects of aberrant proteins, as has been described in NDs or following exposure to EPs, in which case neurodegeneration can be induced [27,28].

In AD, the primary misfolded proteins are Aβ and p-Tau [152]. Disaggregation and degradation of Aβ_1–40_/Aβ_1–42_ and p-Tau proteins are induced by several HSPs [28]. It has been observed that these proteins are refolded by HSP70 and HSP90, which promote their solubility, induce their breakdown through the proteasome system, and reduce their synthesis [35,153,154,155]. Moreover, HSC70, HSP90, and HSP70 coordinate to regulate the polymerization of Tau, preventing its clustering [28,156,157]. It has been shown that overexpression of HSPs or administering extracellular recombinant HSPs decreases Aβ and p-Tau protein concentration and aggregation [28,53,158]. The opposite effect has been observed following the knockdown of HSPs [159]. In this sense, it has been noted that the dysregulation of HSP90 and HSP70 leads to the accumulation and conglomeration of Aβ and p-Tau proteins [49,53]. Additionally, in AD there are several downregulated HSPs, including HSP27, HSP60, HSP70, HSC71, and alpha-crystallin B, which have been described as contributing to this proteinopathy [160,161].

The conformation of Lewy bodies (LB), which lead to neurodegeneration in PD, has been reportedly induced through the accumulation and conglomeration of α-synuclein toxic protein [28]. Several misfolded aberrant proteins, along with α-synuclein and HSPs, are reported to be present in LB [162]. E3-ligase activity, which reduces the deleterious effects of α-synuclein as well as other aberrant proteins through their breakdown, can be induced by HSPs such as HSP70 [163,164]. Otherwise, a decrease of HSP70 protein concentration has been described in the substantia nigra of PD patients [165], and its dysregulation is able to mediate α-synuclein aggregation and conformation of LB [166]. In addition, α-synuclein aggregation has been found to be diminished by several HSPs, including HSP40 and HSP27 [167]. HSC70, which degrades α-synuclein through CMA [168], is reduced in PD [169]. The pathogenesis of PD has been reported to be related to the dysregulation of several HSPs, including HSP90, HSP70, and HSP27 [33].

The accumulation of HTT and the induction of neurodegeneration to which it leads is the primary neuropathological hallmark of HD [28]. The toxicity level of HTT is related to the number of glutamine residues that it contains, becoming more toxic as the residues increase [170]. In HD models, HSP70 has been reported to prevent the induction of toxicity by polyglutamine [171]. The aggregation of polyglutaminated proteins has been described as being prevented by the overexpression of HSP100, HSP70, HSP60, and HSP40, leading to attenuation of disease progression [171,172]. The conformation of HTT has been reportedly reduced by HSP40 and HSP70 [173,174], and it has been shown that CMA, which is induced by several HSPs, eliminates HTT, p-Tau, and Aβ proteins [73]. In addition, it has been suggested that HSP40 dysregulation may lead to toxicity and aggregation of HTT proteins [175]. Likewise, the accumulation of mutant HTT protein aggregates may be induced when HSP40 and HSP70 are dysregulated [176].

Neurodegeneration is induced in PRD when PrPs are accumulated and form prion plaques [28]. PrPs have been found to be reduced by HSP70 through the proteasome system [173], and it has been suggested that HSP100, HSP104, HSP70, HSP42, and HSP26 play a role in avoiding PrP conformation and disaggregation [28,177]. The aggregation and spreading of PrPs is modulated by the dysfunction of several HSPs, as depicted for HSP104 and HSP70 [178].

**Figure 4:**
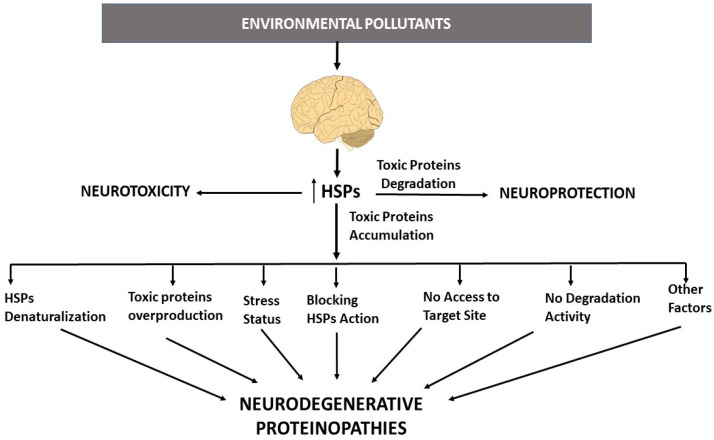
**HSP overexpression and factors that could explain the lack of a neuroprotective effect after EP exposure.** HSP overexpression may not mediate neuroprotective effects or induce neurotoxic effects. Several factors that could mediate the lack of neuroprotective effects of HSPs after EP exposure are shown. Solid line arrows indicate factors and neuroprotective/neurotoxic effects shown in the literature.

However, it is possible that HSP overexpression may not always be able to play a protective role in ND, as their overexpression may not be enough to withstand the excess in aberrant protein accumulation; alternatively, they can be downregulated in a later stage, losing their protective effect, and may even develop harmful activity (Figure 4). Several factors influence the effects of HSPs, including differences in cell type, stress level, gender, and brain region, among others. It has been reported that HSP70 is induced in several NDs, leading to a decrease in aberrant protein levels and neurodegeneration [27,28], while its expression can be decreased with age and brain region in NDs [27]. Moreover, the expression of GRP78 may be differently regulated depending on the stage of illness, gender, brain region, and cell type, as it has been described that its expression is both up- and downregulated in the cortex and hippocampus of AD patients [27,179,180]. Moreover, heme Oxygenase-1 (HSP32) has been described as being induced and playing a role in the degradation of heme into ferrous iron in the neurons and astrocytes of the hippocampus and cortex in AD patients as well as in the astrocytes of the striatum nuclei of PD patients, possibly leading to iron toxicity through the induction of oxidative stress via the Fenton reaction [181,182,183,184]. Although HSP90 has been shown to be induced in several NDs, it has also been shown to be reduced in different brain regions of AD patients [185]. In this sense, it has been observed that HSP90 induction may lead to the formation and aggregation of α-synuclein and its inhibition to a decrease in its generation and deleterious effects [186]. The differences discussed above may be related to the high or low expression of these HSPs in healthy or damaged cells, respectively, and may also be related to the illness stage. HSPs may be better able to compensate for initial/mild damage than high/chronic stress, as they may be disrupted by the latter. Other factors, such as brain region, gender, or environmental factors, may play a role in these differences as well.

Several studies have suggested that neurodegenerative proteinopathies induced by EPs could be mediated through HSP dysfunction, though only a few studies have shown this relationship. In this sense, Al treatment has been reported to downregulate HSPs and increase p-Tau and Aβ protein levels [50], suggesting that the downregulation of HSPs could be the cause of this effect. In another study, As was shown to eliminate the refolding capacity of HSPs, inducing aggregation of toxic proteins [111]. Finally, Cu treatment induced Aβ and α-synuclein proteins through decreased HSPs in IMR cells [46]. Cd, Mn, and PQ single and repeated treatments were reported to induce the formation and accumulation of Aβ_1–40_/Aβ_1–42_ and p-Tau proteins in BF cholinergic SN56 cells and primary hippocampal neurons, which was mediated by HSP downregulation. Co-treatment with these compounds and downregulation of recombinant HSPs partially reversed these effects [49,52,53], proving their mediating effect in the induction of toxic protein accumulation.

## 4. Proteasome and EPs

EPs have been reported to produce proteasome dysfunction and induce toxic protein accumulation [49,51,52,187,188]. However, few studies have established a correlation between EP-induced proteasome dysfunction and EP-induced toxic protein accumulation or proven that the proteasome dysfunction induced by EPs is the cause of toxic protein accumulation. The aim of this section is to discuss this correlation and its causality along with the mechanisms that mediate it.

### 4.1. Environmental Pollutants and Proteasome Dysfunction

EP exposure has been described as inducing ROS, which in turn denaturalize proteins [9,83,125,189] and alter the NRF2 levels [76,77,78,79,80,81,82,83,84,85,86,87,88,89,90,91,92,93] which regulate the expression of proteasome subunits [74]. Thus, proteasome dysfunction can be mediated through NRF2 disruption or the denaturalization of their subunits. However, we cannot discard other mechanisms or direct blockage of its action either.

Several chemical compounds used with different applications have been associated with proteasome dysfunction. Organotins, especially triphenyltin (TPT) and tributyltin (TBT), have been extensively used in antifouling paint for boats, polyvinyl chloride stabilizers, agricultural pesticides, and industrial catalysts. The organotins monophenyltin trichloride (MPT), diphenyltin dichloride (DPT), triphenyltin chloride (TPT), tetraphenyltin (TePT), monobutyltin trichloride (MBT), dibutyltin dichloride (DBT), tributyltin chloride (TBT), and tetrabutyltin (TeBT) have been shown to inhibit P20S activity (chymotrypsin-like activity) after single exposure in several cell lines, resulting in the accumulation of proteasome target proteins and cell death, although no effect was produced on the expression and levels of proteasome subunits [190]. Bisphenol A was reported to downregulate P20S protein levels and inhibit its activity in primary hippocampal cells after single treatment [187]. BPA was shown to decrease NRF2 expression in mice liver [85], which could mediate the decrease of proteasome subunits expression and activity.

Ambient particulate matter (PM) is composed of different smog photochemicals and compounds originating from the combustion of hydrocarbonated substances; 90% of all PM in cities are due to diesel exhaust (DE), and secondary organic aerosols (SOAs) account for 50% of PM in cities [26]. DE and SOAs have been reported to reduce proteasome activity after single treatment of human leucocyte and erythrocyte cells, suggesting that oxidative stress could mediate this effect, although the underlying mechanisms involved in this effect remain unclear [191]. Exposure to cigarette smoke extract in human alveolar epithelial cells results in cell death that is dependent on both time and dosage. This exposure escalates the levels of intracellular ROS, raising the levels of both carbonylated and polyubiquitinated proteins. Furthermore, when both alveolar and bronchial epithelial cells are exposed to high quantities of cigarette smoke extract, all proteasomal functions are hindered, whereas low-concentration exposure impedes the proteasome trypsin-like function [26]. PM and cigarette smoke have been reported to induce and decrease NRF2 expression [192,193,194], which could mediate the effect observed through the alteration of proteasome subunit expression.

Heavy metals impact proteasome function as well [26]. Manganese has been shown to produce P20S inhibition in BF cholinergic SN56 cells after single and repeated treatment [49]. Mn was reported to inhibit P20S activity after single and long-term treatment in PC12 and MES cells [195,196]. However, Mn was reported to present no inhibitory action on P20S activity after single treatment in Neuro-2a, SH-SY5Y, and mouse neural progenitor cells or to inhibit its activity [65,197]. Cd was reported to inhibit P20S activity after single treatment in PC-3 and mouse neural progenitor cells [65,198]. Gallium was reported to inhibit P20S activity after single treatment of several cancer cell lines [199]. Copper was associated with superoxide dismutase 1 (SOD1), α-synuclein, and Aβ protein aggregation [200,201]. Copper was reported to inhibit P20S after single treatment in HeLa cells [202]. Arsenic hampers the function of the proteasome degradation mechanism, and has been found to downregulate the UPS and decrease proteasome activity in several cell lines [203]. Metals, as mentioned previously, have been reported to alter NRF2 levels, meaning that they could alter the expression of proteasome subunits, mediating the above observed effects.

Biocides are associated with proteasome dysfunction as well. In this sense, diethyldithiocarbamate, benomyl, dieldrin, ziram, and endosulfan have been shown to impede proteasome function in SK-N-MCU cells after single and repeated treatment [204]. Additionally, rotenone, paraquat, chlorpyrifos, endosulfan, pendimethalin, fenpyroximate, trichlorphon, tebufenpyrad, and carbaryl have all been reported to induce mitochondrial disintegration, leading to inhibition of proteasome activity after single treatment in SH-SY5Y cells [191]. Exposure of wild-type mice to low doses of maneb, paraquat, and chlorpyrifos has been found to inhibit proteasome complex 26S by downregulating the expression of its subunits [205]. PQ, Maneb, and chlorpyrifos have been reported to alter NRF2 expression [82,83,206], which could mediate the effect observed on proteasome subunit expression.

### 4.2. Proteasome Dysfunction, Neurodegenerative Proteinopathies, and EPs

**Figure 5:**
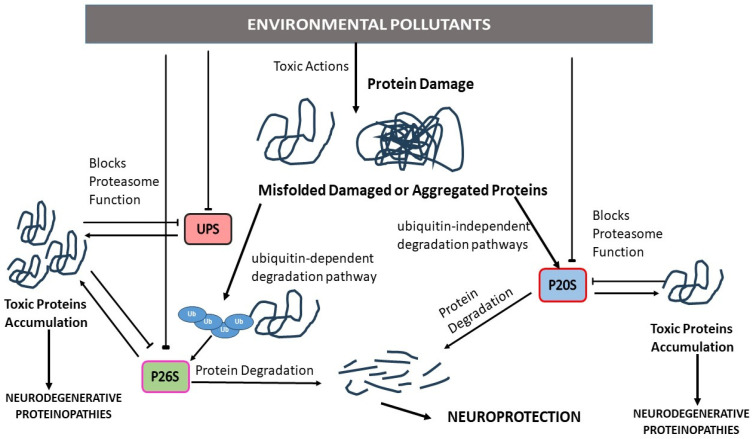
**EP effects on the ubiquitin-dependent and ubiquitin-independent proteasome pathways of protein degradation**. The mechanisms of misfolded toxic protein degradation (ubiquitin-dependent or ubiquitin-independent), their alteration by EP exposure or the accumulation of toxic proteins, and the induction of neurodegenerative proteinopathies. Solid line arrows indicate factors and neuroprotective/neurotoxic effects shown in the literature.

The proteasome plays an important role in the breakdown of unnecessary proteins within cells through both the ubiquitin-dependent and ubiquitin-independent degradation pathways (Figure 5) [31,207]. UPS dysfunction and proteasome activity alteration are related to the accumulation of toxic proteins in several NDs [208,209]. Proteasome activity dysfunction has been described in AD animal models and post-mortem AD patients [210,211,212], showing a region-dependent inhibition effect [210,212]. This effect leads to the accumulation of the toxic proteins Aβ and p-Tau [212,213]. Proteasome action regulates the degradation of the enzymes that mediate Aβ protein synthesis, as its inhibition increases these enzymes and toxic protein levels and its activation reduces their levels [213,214]. The mechanisms that mediate proteasome inhibition are not clear. It has been reported that Aβ and Tau proteins inhibit its activity, which this is supported by a study in which proteasomes were extracted from AD crude lysates. An increase in their activity could be noted based on the absence of toxic proteins [215]. Furthermore, it has been reported that oxidative stress mediates the inhibition of proteasome activity. In this sense, Mn treatment of SN56 basal cholinergic neurons, which is an in vitro model of the basal forebrain in which selective neuronal loss is produced in AD, induces ROS generation that partially mediates proteasome inhibition [49].

UPS dysfunction has been shown to be involved in the pathogenesis of AD [216]. In this regard, parkin, a ubiquitin E3 ligase, is reportedly downregulated in AD, inducing Aβ accumulation [217]; parkin treatment or induction of its overexpression has been described as reversing proteasome dysfunction and Aβ accumulation [217,218]. The UPS enzyme ubiquitin C-terminal hydrolase L1 (UCHL1) is reportedly downregulated in AD [219,220], and its decrease has been shown to induce the accumulation of Aβ [221]. HRD1, a ubiquitin E3 ligase, has been found to be decreased in AD [222]. P-Tau accumulation has been associated with UPS dysfunction as well [223]. However, toxic proteins can induce UPS dysfunction, as described after Aβ treatment in RTN3 transgenic mouse brains, leading to reticulon 3 (RTN3) accumulation in dystrophic neurites [224,225]. Otherwise, although most of the research on UPS malfunction and NDs has focused on neurons, it is important to emphasize that glial cells, which are essential for normal neuronal function, change into a reactive phenotype in NDs, contributing to the development of an inflammatory response that affects UPS function in glial cells. This information highlights the importance of unraveling UPS function in glial cells in NDs [226].

UPS dysfunction and proteasome activity decrease have been reported in the substantia nigra of PD patients. This effect was associated with the decrease of P20S α-subunits levels [227,228,229]. The mechanisms through which the proteasome is inhibited are not well known. Several environmental pollutants (rotenone, paraquat, MPTP, dieldrin, and maneb) which have been suggested as PD etiological factors inhibit the proteasome [212]. Moreover, toxic proteins induced in PD, such as α-synuclein, have been described as inhibiting proteasome activity [230,231]. UPS dysfunction was reported in PD patients, and the dysfunction of its components, such as parkin or UCHL1, leads to α-synuclein accumulation [232].

Several studies have linked UPS and proteasome dysfunction with HD. Proteasome inhibition has mainly been described in the striatum in HD patients, as well as in several HD cell line models [212]. However, no effect was observed in HD mouse models [212]. Proteasome inhibition increases HTT aggregates in cellular models of HD [233]. In addition, proteasome dysfunction has been associated with the accumulation of HTT proteins, which inhibits the proteasome [233]. Conversely, UPS dysfunction does not necessarily have to occur at the proteasome level, and can take place at any level of the system, such as the ligation process. This is facilitated several E3 ligases that have been shown to ubiquitylate HTT protein, including WWP1 [234], UBE3A [235], HACE1 [236], CHIP [237,238], HRD1 [239], and Parkin in mammals [240], as well as LTN1in yeast [239,241]. Although these studies support the hypothesis of a relationship between UPS impairment and HD, it is difficult to draw a conclusion, as contradictory results have been reported.

Proteasome dysfunction has been reported in ALS, with inhibition of the proteasome in the spinal cord and a decrease in proteasome β5 subunit levels [212]. These effects have been shown in mouse models of ALS, with region-dependent proteasome activity and expression decrease observed [212]. Several studies have reported that SOD1 accumulation in mouse models of ALS is produced by proteasome inhibition and that its inhibition produces greater damage in ALS mouse models [228]. Additionally, TDP-43 protein was reportedly accumulated in ALS, with the suggestion that this effect is produced by proteasome inhibition [212].

EP exposure has been associated with the induction of proteinopathies through proteasome inhibition [49,51,52,187,188]. Dieldrin, rotenone, paraquat, and diethyldithiocarbamate exposure have been reported to induce α-synuclein fibril formation or to increase α-synuclein levels, suggesting a possible mediation of this effect through proteasome dysfunction [54,55,64]. In this regard, dieldrin has been shown to induce the aggregation of α-synuclein and proteasome inhibition [242]. Paraquat increases phosphorylated-Tau and α-synuclein levels and decreases proteasome activity in the striatum of mice repeatedly exposed [54], suggesting that this effect could be mediated by proteasome dysfunction as well. Cd single treatment induced Aβ proteins accumulation through P20S inhibition in BF SN56 cholinergic neurons [243], and Cd single treatment was shown to induce the formation of prion proteins through proteasome inhibition in mouse neuronal cells [65]. Moreover, Mn, chlorpyrifos, and PQ have been reported to induce accumulation of p-Tau and Aβ proteins through reduced proteasome 20S activity after 1 day and 14 days of treatment in SN56 cells and primary hippocampal neurons [49,51,52], showing the participation of proteasome dysfunction in the accumulation of aberrant toxic proteins.

## 5. Therapeutic Strategies against Proteinopathies

Different therapeutic strategies have been developed to treat the neurotoxic effects induced by proteinopathies. In this sense, antibody therapy has been developed against specific toxic proteins (Aβ, tau, HTT, and α-syn) by dissolving and removing their aggregates [24,244]. Another therapeutic approach is the use of antisense oligonucleotides for the silencing toxic protein synthesis [24]. However, these therapies have the drawback of crossing the blood–brain barrier (BBB) and acting on specific brain regions and cells [245]; most importantly, they can only act on one type of toxic protein.

**Figure 6:**
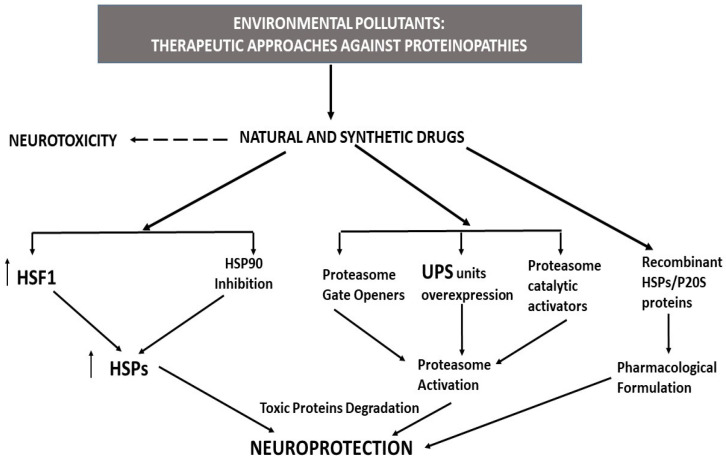
**HSPs and proteasome as therapeutic tools against metal EP-induced neurodegenerative proteinopathies.** Several strategies involving the use of HSPs/proteasome recombinant proteins or the induction of their overexpression/activation have been developed as therapeutic approaches. Drugs that inhibit HSP90 or induce HSF1 expression have been developed to upregulate HSPs. Drugs that open the proteasome gates or induce its overexpression and catalytic activation have been developed to activate the proteasome activity. Solid line arrows indicate mechanisms of HSP/proteasome overexpression/activation and neuroprotective effects shown in the literature. The dashed line arrow shows the possible toxic effects of the developed drugs that need to be evaluated.

NDs are characterized by being mediated through more than one known toxic protein, and possibly others that are unknown, requiring a therapeutic strategy to target the removal of all types of toxic proteins present in the cells. several strategies have been developed to address this issue, including gene expression upregulation, activation, or administration of recombinant HSPs and/or P20S proteins (Figure 6) [24,49,52,53,245,246,247].

### 5.1. HSP Activators/Inductors as Therapeutic Tools for the Neurodegenerative Proteinopathies Induced by Environmental Pollutants

EP-induced stress has been shown to be reduced through HSPs as a protective mechanism, and their overexpression has been shown to reduce the deleterious effects of EPs [27,35,158]. Therefore, research has been developed to find methods of HSP overexpression induction to reduce both the harmful effects of NDs and the neurodegenerative effects of EPs (Figure 6). HSP expression can be induced through an increase in HS1 levels [53,248] or by the inhibition of HSP90, as HSP90 sequesters HSF1 in the cytosol; when HSP90 is inhibited, HSF1 is released and migrates to the nucleus, leading to the induction of HSP expression [170,248].

Different natural compounds such as geldanamycin, carbenoxolone, celastrol, curcumin, gambogic acid, withaferin-A, jujuboside-A, paeoniflorin, myricetin, quercetin, glycyrrhizin, geranylgeranylacetone, resveratrol, capsaicin, and ginkgo biloba, among others, have been shown to induce the expression of HSPs such as HSP70, HSP40, HSP27, HSP30, and others through HSF1 upregulation, HSP90 inhibition, or both [28,50,247,249,250,251,252,253]. Additionally, synthetic compounds have been tested for HSP induction ability: 17-allylamino-17-demethoxy-geldanamycin (17-AAG), 17-dimethylaminoethylamino-17-demethoxygeldanamycin (17-DMAG), SNX compounds, bimoclomol (BRLP-42), rebamipide, LA1011, PNU282987, BIIB021 (CNF-2024), ASS234, and 7-amino-phenanthridin-6(5H)-one derivatives (APH) have all been shown to upregulate HSP expression [50,100,242,246,254,255,256,257,258]. ASS234 was shown to induce HSF1 as well as expression of several other HSPs (HSP105, HSP90AB1, HSPA1A, HSPA1B, HSPA5, HSPA8, HSPA9, HSP60, DNAJA1, DNAJB1, DNAJB6, DNAJC3, DNAJC5, DNAJC6, HSPB1, HSPB2, HSPB5, HSPB6, HSPB8, and HSP10) in SH-SY5Y cells, probably through HSF1 upregulation [100]. APH compounds have been reported to increase HSP70 expression, and Cd co-treatment with different APHs compounds was found to reverse Cd-induced Aβ protein aggregation and SN56 cell death through HSP70 upregulation [259]. These compounds have been shown to reduce the accumulation and aggregation of toxic proteins such as Aβ, Tau, α-synuclein, and HTT, among others, through HSP upregulation, thereby improving ND [28,259].

However, there have been several cases in which the compounds induced toxicity, such as the case of GA and 17-AAG [260,261], or generated greater damage following HSP overexpression, as happened with celastrol, in which case more cell death was observed due to HSP70 overexpression instead of a protective effect on primary motoneurons against H_2_O_2_ or staurosporine-induced apoptosis [261]. Furthermore, it has been reported that the overexpression of HSP70 did not induce any neuroprotective effect in epilepsy [262,263]. Thus, in each pathological condition, the HSPs being altered should be induced specifically to prevent adverse effects. Otherwise, the use of specific recombinant HSPs as therapeutic tools for particular pathologies [158] has been suggested, as extracellular HSPs are secreted by several non-neuronal cells for neuronal protection [264,265,266].

### 5.2. Proteasome Activators as Therapeutic Tools for Neurodegenerative Proteinopathies Induced by Environmental Pollutants

The proteasome is necessary for the clearance of damaged or misfolded toxic proteins; however, in NDs and after environmental pollutant exposure its activity declines [30,191,204,267]. Thus, proteasome activation or induction has been explored as a new field in ND drug development and treatment of contaminant-induced neurodegenerative proteinopathies. Different mechanisms which are able to increase proteasome activity have been reported. In this respect, denaturation of the proteasome, facilitating access of target proteins to the catalytic pocket by opening the gate of proteasome α-ring, inducing overexpression of the proteasome subunits, and stimulation of catalytic activity have all been reported as possible therapeutic approaches [268].

Denaturalizing compounds such as sodium dodecyl sulfate or poly-lysine have been reported to increase the activity of the proteasome, which is suggested to be mediated through partial denaturalization of the α-ring subunits [268]. However, these types of compounds are not being used currently, as it is not possible to obtain specific interactions and due to the difficulties involved in their optimization [269]. Phenothiazines such as chlorpromazine and imidazolidines such as TCH-165, as well as modified analogs designed to increase proteasome activity, increase chymotryptic-like activity by opening the P20S α-ring gate, preventing the accumulation of several toxic proteins [268]. In addition, synthetic peptides have been developed which are able to open the gate of the P20S α-ring, thereby increasing proteasome activity [268]. In this sense, the proteasome-activating peptide 1 (PAP1), which increases proteasomal activation by opening the proteasomal catalytic chamber and preventing SOD1 aggregation in an ALS cell line model, has recently been developed [270]. However, this strategy is not appealing, as the use of peptides presents problems such as high metabolization rate and difficulties with membrane trespassing, among others, limiting development of synthetic peptides [268].

Proteasome activation has been carried out by increasing catalytic processing, inducing kinase phosphorylation in specific sites of P26S. Multiple P26S phosphorylation sites (>455) which modulate its assembly, stability, and activity have been identified [271]. Rolipram, a small molecule inhibitor of phosphodiesterase type 4 (PDE4), induces phosphorylation of the P19S Rpt6 subunit by cAMP-dependent protein kinase A (PKA), which upregulates P26S assembly and proteasome activity in vitro (cAMP-induced phosphorylation of P26S proteasomes on Rpn6/PSMD11 enhances their activity and the degradation of misfolded proteins). Rolipram promoted the clearance of abnormal p-Tau and improved cognition in a mouse model of AD [272].

NRF2 factor has been shown to induce the upregulation of proteasome subunits [268]. In this regard, 3H-1,2-dithiole-3-thione (D3T) was shown to produce P19S and P20S overexpression in fibroblast and prevent Aβ peptides accumulation, reducing cognitive decline induction in a mouse AD model [268]. Sulforaphane, tert-butylhydroquinone, and 18α-glycyrrhetinic acid (18α-GA) have been reported to induce proteasome subunit overexpression as well [268]. Finally, other compounds with unknown mechanisms that mediate the induction of proteasome activity have been identified, including pyrazolones and PD169316, a p38 MAPK inhibitor which has been found to reduces α-synuclein levels in primary mouse neurons [268].

Several natural compounds have been identified as P20S activators or inductors as well. In this sense, betulinic and ursolic acids, both of which are triterpene compounds, have been described as activating P20S [268]. Betulinic acid mediates the action of P20S by increasing chymotryptic-like activity through the P20S α-ring gate opening [273]. Several polyphenols, such as epigallocatechin-3-gallate (EGCG) found in green tea and carnosic acid found in rosemary, have been shown to enhance P20S activity [274,275]. These compounds increase P20S chymotryptic-like activity, for instance through parkin upregulation in the case of carnosic acid, preventing the accumulation and aggregation of toxic proteins [275,276,277]. Flavonoids such as quercetin and fisetin have been reported to induce the activation of proteasome activity as well, leading to the degradation of toxic proteins [278,279,280]. Quercetin has been reported to increase proteasome activity through NRF2 upregulation, leading to proteasome subunit overexpression [278].

### 5.3. Recombinant HSPs and P20 Proteins as Therapeutic Tools against Neurodegenerative Effects Induced by Environmental Pollutants

Recombinant HSPs and P20S proteins have been studied as possible therapeutic agents for neurodegenerative processes (Figure 6). Intranasal treatment with recombinant HSP70 (rHSP70) in a transgenic mouse model of AD reportedly decreased the accumulation of Aβ proteins and prevented neurodegeneration in the hippocampus and cortex [265]. ROS generation, Aβ, and α-synuclein aggregation, and cell death induced following Cu exposure to IMR-32 human neuroblastoma cells were all reversed following rHSP27 co-treatment with Cu for 24 h [46], highlighting its therapeutic ability against Cu toxicity. The effect of recombinant HSPs and P20S proteins against EP-induced toxicity in basal forebrain SN56 cholinergic neurons and primary hippocampal neurons was studied in [49,51,52,53,258]. Aβ and p-Tau protein accumulation following Cd exposure was partially reversed following 24 h of Cd and recombinant P20S (rP20S) protein exposure in SN56 cells [258]. Cd co-treatment for 24 h with recombinant HSF1 partially reversed Cd-induced HSF1 downregulation that led to HSP90α, HSP701A, and HSP27 expression being decreased in SN56 cells [53] The increase in Aβ and p-Tau proteins induced by Cd treatment in SN56 cells was reduced following 24 h Cd co-treatment with recombinant HSP90 (rHSP90), HSP70 (rHSP70), and HSP27 (rHSP27) either independently or simultaneously [53]. The reduction in the levels of these proteins was greater under Cd co-treatment with recombinant HSF1 (rHSF1) than under simultaneous co-treatment with rHSP27, rHSP70, and rHSP90, indicating that other HSPs are involved in this effect, as HSF1 downregulation is able to induce the suppression of several HSPs [53]. P20S and the HSPs have been shown to have a neuroprotective effect against the toxic effects of p-Tau and Aβ proteins, as recombinant co-treatment with Cd partially reversed the cell loss induced following Cd treatment in SN56 cells [53,258]. Lastly, Cd and rHSF1 concurrent treatment induced a lesser reversion of the cell death compared to that induced following Cd treatment of concurrent βAPP and Tau knockdown cells; thus, it appears that further toxic proteins may be involved in the toxic effects of Cd [53].

HSP90 and HSP70 levels and P20S activity were decreased following Mn treatment on SN56 cells for 24 h and for 14 days [49]. Aβ and p-Tau proteins accumulation following one- or fourteen days of Mn treatment was reversed in part through Mn co-treatment with rHSP90, rHSP70, and rP20S [49]. The induced apoptosis was reversed in part after Mn and recombinant protein treatment in SN56 cells [49]. Chlorpyrifos (CPF) treatment for 24 h and 14 days decreased proteasome 20S activity which led to a rise in toxic proteins such as p-Tau and Aβ and the induction of apoptotic cell death in SN56 cells, which was partially reversed after CPF co-treatment with rP20S [51]. PQ alters HSP70 expression, partially mediating p-Tau and Aβ protein accumulation that otherwise led to rat primary hippocampal neuronal cell death following 1 day and 14 days of treatment [52]. PQ co-treatment with rHSP70 of primary hippocampal neurons partially reversed the accumulation of Aβ and p-Tau proteins and the neuronal loss observed after PQ treatment alone [52]. These data suggest that recombinant HSPs and P20S proteins are a very promising alternative to treat neurodegeneration induced by different toxic stimuli. However, this therapeutic approach is inconvenient, as it is not possible to administer these proteins systemically. Studies have not yet detected a significant presence in the brain after their administration, making intranasal or local intracranial administration necessary in order to ensure their presence and protective results [158].

Only small lipophilic molecules can cross the BBB freely, while hydrophilic or large molecules are impeded from crossing it [46]. Endogenous large molecules needed for brain function can cross the BBB through specific membrane-like insulin receptor (IR), transferrin receptor (TfR), insulin-like growth factor receptor (IGFR), leptin receptor (LPR), low-density lipoprotein receptor (LDLR), and Fc receptor (FcR), among others [46,248]. Recombinant proteins and many new therapeutic molecules cannot cross the BBB and access the brain to mediate their therapeutic effect; thus, different strategies have been developed to solve this problem. One strategy is based on the natural transport mechanisms of the large molecules discussed above, by conjugating the recombinant protein or drugs with one of the natural peptides that present transport receptors or by conjugating these molecules to monoclonal antibodies (MAb) that recognize and bind some of these receptors to cross the BBB [46,246]. The primary MAb employed to date recognizes the human insulin receptor (HIRMAb); an example is the iduronidase lysosomal enzyme (IDUA) fused to HIRMAb, forming HIRMAb-IDUA, which is used to treat mucopolysaccharidosis Type I, as UDUA is inactive due to its mutation in this disease [46,246].

Another strategy is to use nano-pharmaceutical formulations, which can allow these molecules to cross the BBB and reach the specific brain locations where the drugs are needed, thereby reducing the dosage frequency and toxicity [281,282]. Several types of formulations are used for ND treatment, including liposomes, lipid systems, polymeric nanoparticles, magnetic nanoparticles, and dendrimers, among others [281,282,283].

## 6. Future Directions

There is increasing evidence pointing out a relationship between environmental pollutants and proteinopathy diseases, highlighting the need to develop further studies researching the involvement contaminants in disease induction and development as well as the mechanisms through which HSPs and proteasome dysfunction mediate its induction. Contaminants with highly different structures are able to induce the same proteinopathies and neurodegenerative effects, making it important to understand the mechanisms through which they mediate these effects in order to improve treatment of proteinopathy-neurodegenerative diseases.

Contradictory effects on HSPs have been described after contaminant exposure, highlighting the need to perform further studies in order to elucidate the reasons for these opposite effects and the relevance of different facts such as time of exposure, dose, and gender, among others, as well as which specific HSPs are altered in the brain. Moreover, it should be determined whether the reduction in proteasome activity observed in NDs and after environmental contaminant exposure is mediated by the direct action of EPs or through indirect action due to the accumulation of toxic proteins. Proteasome regulation is very complex, and it is necessary to understand its complete regulation in order to aid in the development of new, efficient, and specific drugs for the treatment of proteinopathies and neurodegenerative diseases induced by EPs. Such data would lead to a better management and treatment of EP-induced proteinopathies.

However, several of the drugs developed to date have been shown to present toxicity, and others could potentially induce it. One example of this occurs with NRF2 activators, as proteasome inductors may be able to induce cell proliferation, making them possible tumor inductors [270]. Proteasome activators have been described as inhibiting autophagy, with non-predictable final results on proteostasis effects [270]. Moreover, no long-term studies have been performed on these compounds, and further safety studies on these drugs need to be developed.

Recombinant HSPs and P20S appear to be promising non-toxic tools for global treatment of proteinopathies. However, combined treatment with recombinant HSPs and P20S seems to not completely compensate for the effects induced by toxic proteins [49,52], indicating that other mechanisms could be induced, such as autophagy dysfunction, disruption of other HSPs, or different alterations to the UPS. Moreover, recombinant proteins are not properly delivered to the brain through the BBB. Therefore, further studies should be developed to determine this information and explore different mechanisms to improve recombinant protein delivery to the nervous system.

Addressing all these issues will allow for a complete understanding of these contaminants’ effects on HSPs and the proteasome system, as well as the associated factors that could modify these effects. These developments will allow appropriate calculation of risk following contaminant exposure, help to establish protective actions, and a present more efficient therapeutic approach.

## 7. Conclusions

Several NDs, including AD, ALS, MS, PD, and PRD, among others, have been suggested to have EPs as possible etiological factors. Dysregulation of the HSP family and decreased proteasome activity, along with the resulting inability to eliminate the high levels of accumulated toxic proteins, appear to mediate the induction of neurodegenerative disorders and neurodegeneration produced after EPs exposure. There is a bidirectional relationship between the accumulation of aberrant proteins and the dysfunction of HSPs and the proteasome system, which together lead to cell damage and loss. Increased proteasome activity and HSP overexpression prevent the accumulation of aberrant proteins and the cell damage induced by them. Therefore, several therapeutic approaches have been developed to achieve this effect, although a number of the drugs designed to date induce toxic effects, and insufficient drug safety studies have been carried out. A possible therapeutic strategy which may have better outcomes and avoid toxicity could be the treatment of individual NDs with specific recombinant proteins for the involved downregulated HSPs together with recombinant P20S. However, new recombinant protein formulations which allow for adequate delivery to the brain need to be developed. Further studies are needed to clarify the role of EPs on HSPs and proteasome dysfunction, as well as to develop more efficient drugs to prevent and treat neurodegenerative proteinopathies.

## Data Availability

Not applicable.
